# Supplementary material for: Fabrication and evaluation of complicated microstructures on cylindrical surface
Source: PLoS One. 2020 Dec 15;15(12):e0242918. doi: 10.1371/journal.pone.0242918 (PMC7737994; doi:10.1371/journal.pone.0242918)
Supplement: S1 File — (DOC) [file pone.0242918.s001.doc]

**Algorithm programming realization**

There is a certain spatial relationship between the microstructure array units, which can be described by mathematical methods. Writing a processing program for each microstructure unit is usually only effective for a small number of arrays. When a large number of microstructures need to be processed, it is an effective programming method to use the spatial relationship between the microstructure units. Take the regularly distributed spherical microstructure as an example, the process of programming is following

The circular grating of the C axis outputs pulse signals at equal angles, and the tool path for cylindrical microstructure processing is actually an equidistant spiral.

As shown in S1.Fig 1(A), the radius of workpiece is *R*, the Circumference is *C*, and the machining length is *L*. Supposing the diameter of each spherical surface is d and there are *m*×*n* microstructure units, then the axial cycle is *L*/*m*, and the Circumferential cycle is *C*/*n*.

The steps of programming are as follows

(a) The processed area is C×*L*, and the mesh is divided in this range to obtain the micro-structured mesh unit (*L*/*m*×*C*/*n*).

(b) Convert cylindrical coordinate information into rectangular coordinate information

(*θ,R,z*)→(*z,y,x*).

Where *y* = *Rθ* (0≤*θ*≤2π), *θ* and z are Z-axis position signal and C-axis angle signal obtained from UMAC.

In the ZOY plane, The coordinate of the tool center is A(*z,y*)

(c) Judge whether the tool center is in the microstructure processing area, if not, the controller output voltage is 0V. If already in the area, proceed to the next step.

(d) The center coordinates (*l_i_,s_j_*) of the corresponding microstructure units is obtained by the Z-direction period *i* and Y-direction period *j* of point A (*z, y*) with Eq S.1

$l_{i}=\frac{d}{2}+i\frac{L}{m},s_{j}=\frac{d}{2}+j\frac{C}{n},\left(i=0,1,...m-1,j=0,1,...n-1\right)$ (S.1)

(e) Judge whether (*z, y*) is within the range of machining aperture *d*. If yes, then calculate tool radius compensation according to Eq S.2, otherwise the displacement is 0μm.

$x_{1}=f\left(z_{1}-\frac{r_{\varepsilon}\cdot\frac{\partial f}{\partial z_{0}}}{\sqrt{1+\left(\frac{\partial f}{\partial z_{0}}\right)^{2}}},\theta\right)-\frac{r_{\varepsilon}}{\sqrt{1+\left(\frac{\partial f}{\partial z_{0}}\right)^{2}}}$ (S.2)

(f) After the tool radius compensation value is obtained, the feed amount in the X direction is obtained, which is converted into the corresponding output voltage by the voltage-displacement proportional coefficient k of the fast tool servo system.

(g) Return to step (b) and calculate the next point.

S1.Fig 1(B) is the flow chart of processing program.


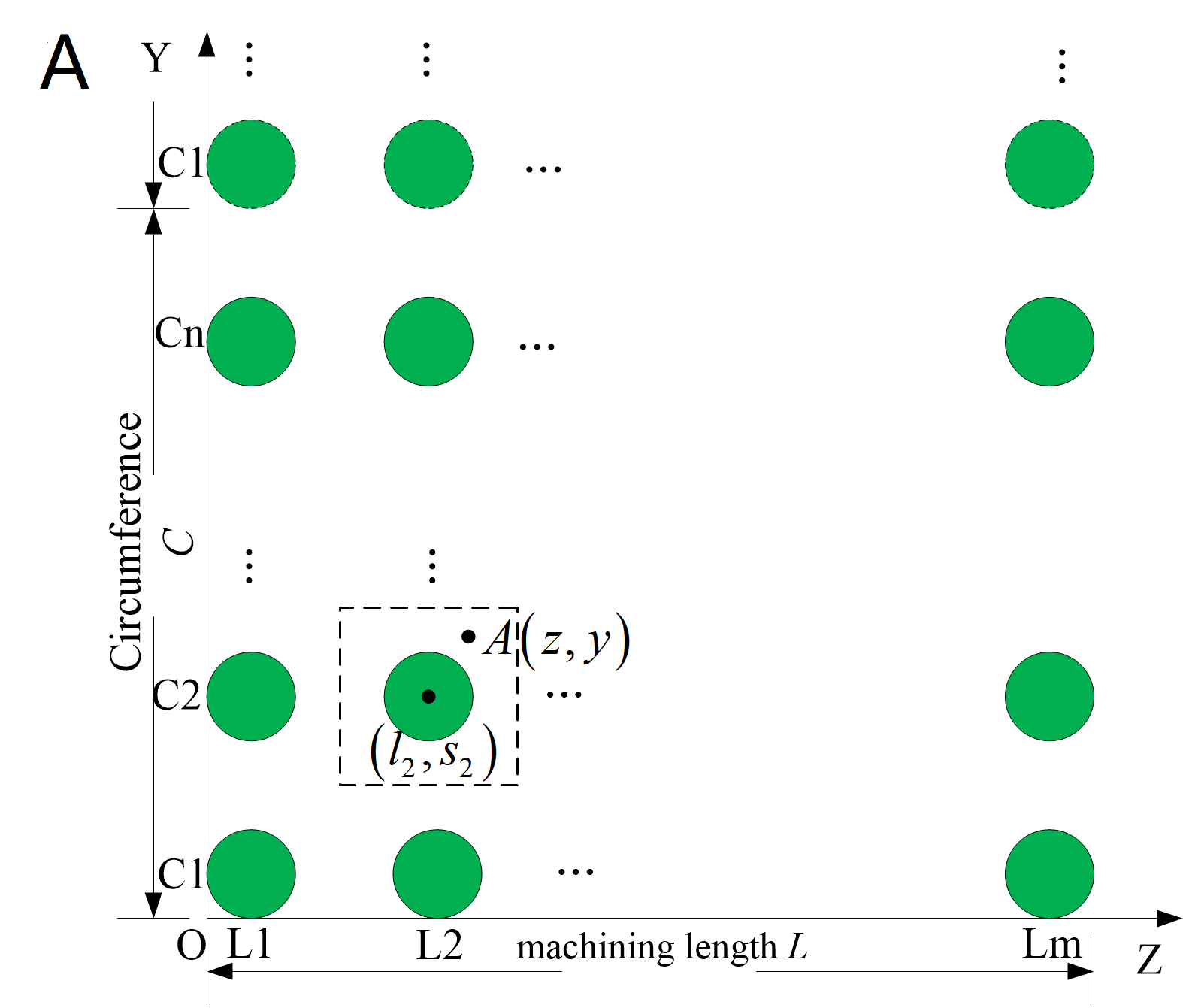

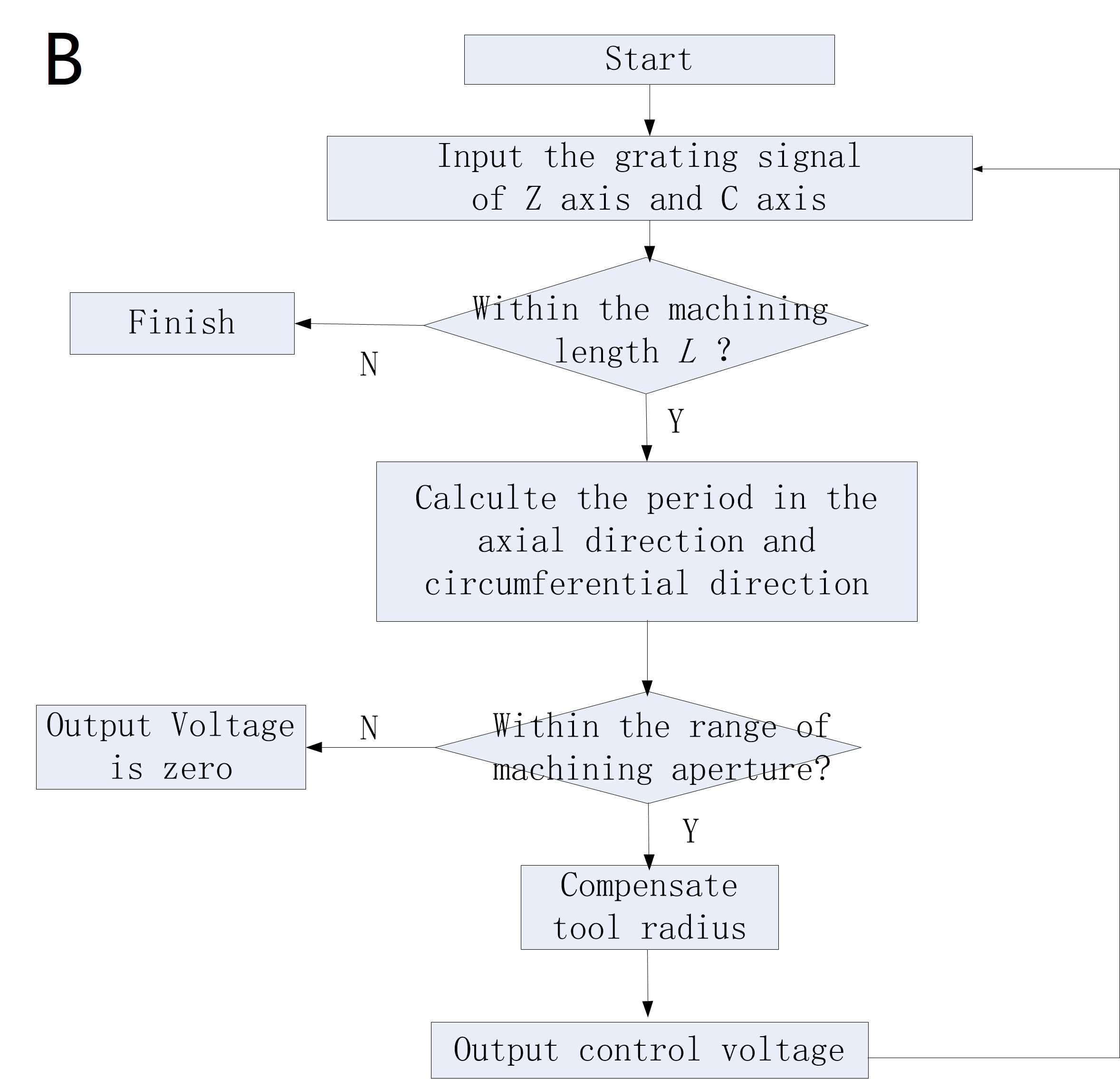


**S1. Fig 1. Spherical microstructure array and its programming process.** (A) Spherical microstructure array (B) Programming process
