# Supplementary material for: Fabrication and evaluation of complicated microstructures on cylindrical surface
Source: PLoS One. 2020 Dec 15;15(12):e0242918. doi: 10.1371/journal.pone.0242918 (PMC7737994; doi:10.1371/journal.pone.0242918)
Supplement: Attachment — Submitted filename: Response to Reviewers.docx [file pone.0242918.s002.docx]

Journal Requirements:

When submitting your revision, we need you to address these additional requirements

1. Please ensure that your manuscript meets PLOS ONE's style requirements, including those for file naming. The PLOS ONE style templates can be found at https://journals.plos.org/plosone/s/file?id=wjVg/PLOSOne_formatting_sample_main_body.pdf and https://journals.plos.org/plosone/s/file?id=ba62/PLOSOne_formatting_sample_title_authors_affiliations.pdf

2.We suggest you thoroughly copyedit your manuscript for language usage, spelling, and grammar. If you do not know anyone who can help you do this, you may wish to consider employing a professional scientific editing service.  

Upon resubmission, please provide the following:

- The name of the colleague or the details of the professional service that edited your manuscript
- A copy of your manuscript showing your changes by either highlighting them or using track changes (uploaded as a *supporting information* file)
- A clean copy of the edited manuscript (uploaded as the new *manuscript* file)

Response: We regret there were problems with the English. The paper has been carefully revised by a professional language editing service from LetPub to improve the grammar and readability. LetPub is an author service brand owned and operated by Accdon LLC. Headquartered in the Boston area, It is a full-spectrum author services company with a large team of US-based certified language and scientific editors, ISO 17001 accredited translators, and professional scientific illustrators and animators.

3. PLOS requires an ORCID iD for the corresponding author in Editorial Manager on papers submitted after December 6th, 2016. Please ensure that you have an ORCID iD and that it is validated in Editorial Manager. To do this, go to ‘Update my Information’ (in the upper left-hand corner of the main menu), and click on the Fetch/Validate link next to the ORCID field. This will take you to the ORCID site and allow you to create a new iD or authenticate a pre-existing iD in Editorial Manager. Please see the following video for instructions on linking an ORCID iD to your Editorial Manager account: https://www.youtube.com/watch?v=_xcclfuvtxQ

 Response:The ORCID iD of the corresponding author is 0000-0002-7089-2388. I have updated my information in Editorial Manager.

4. We note you have included a table to which you do not refer in the text of your manuscript. Please ensure that you refer to Table 1 in your text; if accepted, production will need this reference to link the reader to the Table.

Response: Table 1 referred in this paper was written as Figure 1, which has been corrected.

[Note: HTML markup is below. Please do not edit.]

Reviewers' comments:

Reviewer's Responses to Questions

**Comments to the Author**

1. Is the manuscript technically sound, and do the data support the conclusions?

The manuscript must describe a technically sound piece of scientific research with data that supports the conclusions. Experiments must have been conducted rigorously, with appropriate controls, replication, and sample sizes. The conclusions must be drawn appropriately based on the data presented.

Reviewer #1: Partly

Reviewer #2: Partly

2. Has the statistical analysis been performed appropriately and rigorously?

Reviewer #1: N/A

Reviewer #2: No

3. Have the authors made all data underlying the findings in their manuscript fully available?

The [PLOS Data policy](http://www.plosone.org/static/policies.action#sharing) requires authors to make all data underlying the findings described in their manuscript fully available without restriction, with rare exception (please refer to the Data Availability Statement in the manuscript PDF file). The data should be provided as part of the manuscript or its supporting information, or deposited to a public repository. For example, in addition to summary statistics, the data points behind means, medians and variance measures should be available. If there are restrictions on publicly sharing data—e.g. participant privacy or use of data from a third party—those must be specified.

Reviewer #1: No

Reviewer #2: Yes

4. Is the manuscript presented in an intelligible fashion and written in standard English?

PLOS ONE does not copyedit accepted manuscripts, so the language in submitted articles must be clear, correct, and unambiguous. Any typographical or grammatical errors should be corrected at revision, so please note any specific errors here.

Reviewer #1: No

Reviewer #2: Yes

5. Review Comments to the Author

Please use the space provided to explain your answers to the questions above. You may also include additional comments for the author, including concerns about dual publication, research ethics, or publication ethics. (Please upload your review as an attachment if it exceeds 20,000 characters)

Reviewer #1: 1. Is the manuscript technically sound, and do the data support the conclusions?

In general, the various technical parts that make up the method seem sound (compensation of tools radius, optimization of the hinge parameters). Most of the methods used to solve them are standard practice (e.g. bilinear interpolation).
However, certain aspects are not clear:

- Could you clarify if the derivation of $\sigma$ is analogous the $S$ (Equations 2 and 3), such that both quantities are comparable?

Response: Response: Stress S andσ strength are functions of many random variables, such as material properties, size parameters and environmental variables. The degree function is very difficult to obtain directly. It is necessary to synthesize the distribution of relevant parameters into the distribution of stress and strength Including algebraic method, moment method and Monte Carlo method

- Function $f$ in Equation 2 is the same as function $f$ in Equation 1? Shouldn't it be the inverse function since one reads $f(S) and the other $S=f(x1,x2,...xn)$?
Response: They are not a function, the probability density function of stress in equation (1) and the variable function of stress based on moment method in equation (2).

- Equation 3: how are the second and first order differentials calculated?
Response: It can be obtained by algebraic method or moment method.

- Line 122: another different function named $f$, I recommend to change the nomenclature. Could you also precise what $z$ and $\theta$ are in this function?
Response: The terminology and supplementary instructions have been changed as required by the reviewer.

- Line 127: could you define beforehand what is "section curve" and "tool center path"?
Response: Pre description has been made in accordance with reviewer opinion.

- Equation 7: another function named $f$ which I assume is different from the previous?

Response:They are different, and we changed the function symbol.

The precision of the method is backed up with comparisons between the ground truth and the result considering some error metrics (section "Precision evaluation of microstructure arrays"). The results seem to indicate that the procedure decreases the error of the fabrication process.
Response: In this chapter, the simulation results are compared, and then the evaluation method is applied to the later processing evaluation.

2. Has the statistical analysis been performed appropriately and rigorously?

N/A.

3. Have the authors made all data underlying the findings in their manuscript fully available?

I was unable to find the data related to the different plots. Also, It would be appreciated if the authors made the code of the "compensation algorithm" available as well.
Response: We will upload the compensation algorithm programming steps as supplementary materials. The filename is S1_Compensation Algorithm.

4. Is the manuscript presented in an intelligible fashion and written in standard English?

The descriptive and technical content of the manuscript is often difficult to follow, mostly due to unusual phrasing and grammar.
Authors should seek help in correcting and polishing the text, otherwise, it becomes sometimes an uphill task to read it.

Besides, it would be appreciated it the main goal of the manuscript is described in more detail in the introduction section, along with the corresponding challenges.

Response:It has been revised according to the reviewer' opinions. We regret there were problems with the English. The paper has been carefully revised by a professional language editing service from LetPub to improve the grammar and readability. LetPub is an author service brand owned and operated by Accdon LLC. Headquartered in the Boston area, It is a full-spectrum author services company with a large team of US-based certified language and scientific editors, ISO 17001 accredited translators, and professional scientific illustrators and animators.

Reviewer #2: This paper presents a pipeline to optimize the machinability of the FTS system used to fabricate microstructures on roll molds. The authors first optimize the reliability of the fabrication system by selecting a proper design parameter of a flexible hinge holder. A tool radius compensation method is used to enhance the fabrication precision, where the least-square solution is used to approximate the cut depth. To verify the fabrication result, an evaluation method is generated to reduce the evaluation error.

I believe the contribution of this work is solid as the precision of the final fabricated microstructure is greatly enhanced and seems aspheric surface is successfully fabricated. Although it needs to be mention that the tool radius compensation method used in the paper is not novel in the precision fabrication field. The manuscript is also not well-organized, and I have the sense that each part is weekly connected and not properly justified. My main concern is that most figures are not well prepared that cannot give enough information to support their conclusions. Detailed comments are as follows.

* Line 80, please add the description of where the hinge holder is used in the FTS system (better to add a figure to illustrate the fabrication process). And please make it clear how the FEM simulation is applied to the component and describe how the boundary condition and workload are set.

Response: Relevant supplementary explanations have been made according to the advice.

* The resolution of Fig 4 is very low, and the data is the loss of the unit.

Response: Figure 4 is the cloud image saved by ANSYS software, which is already the highest resolution.

* Line 137, why the aspheric is hard to be obtained by Eq.6? Since designed aspheric surfaces can always have an implicit function to explain the surface and used in parameterization function.
Response: The expression in the text is not accurate, and the meaning of the expression has been revised according to the suggestion

* The section “precision evaluation of microstructure arrays” is hard to follow. Please first add the explanation where the evaluation error comes from.

Response: Relevant supplementary explanations have been made according to the advice.

* The captain of Fig 6 cannot match with the figure itself.
Response: The captain of Fig 6 is corrected according to the advice.

* Fig 8, the target aspheric structure should be added as a ground-truth for reference.

Response: Figure 8 shows the simulation diagram before and after the tool radius compensation. If you refer to figure 9, the first picture has been taken on the blue tape desktop to facilitate the observation of microstructure

* Fig.12 basically gives no useful information as the ground-truth of the surface is not shown. Please also add a reference distance scale in the figure.
Response: Figure 12 shows wear, which has been remarked.

In summary, I do think this paper requires heavily modify before it is ready for publication. The author should make the paper well organized and make all the figures easy to understand and technical details clear to make their claim self-contained.

6. PLOS authors have the option to publish the peer review history of their article ([what does this mean?](https://journals.plos.org/plosone/s/editorial-and-peer-review-process#loc-peer-review-history)). If published, this will include your full peer review and any attached files.


If you choose “no”, your identity will remain anonymous but your review may still be made public.


**Do you want your identity to be public for this peer review?** For information about this choice, including consent withdrawal, please see our [Privacy Policy](https://www.plos.org/privacy-policy).

Reviewer #1: No

Reviewer #2: No
