# Supplementary material for: Fabrication and evaluation of complicated microstructures on cylindrical surface
Source: PLoS One. 2020 Dec 15;15(12):e0242918. doi: 10.1371/journal.pone.0242918 (PMC7737994; doi:10.1371/journal.pone.0242918)
Supplement: Attachment — Submitted filename: Response to Reviewers.docx [file pone.0242918.s003.docx]

**Comments to the Author**

1. If the authors have adequately addressed your comments raised in a previous round of review and you feel that this manuscript is now acceptable for publication, you may indicate that here to bypass the “Comments to the Author” section, enter your conflict of interest statement in the “Confidential to Editor” section, and submit your "Accept" recommendation.

Reviewer #1: All comments have been addressed

Reviewer #2: (No Response)

2. Is the manuscript technically sound, and do the data support the conclusions?

The manuscript must describe a technically sound piece of scientific research with data that supports the conclusions. Experiments must have been conducted rigorously, with appropriate controls, replication, and sample sizes. The conclusions must be drawn appropriately based on the data presented.

Reviewer #1: Yes

Reviewer #2: Yes

3. Has the statistical analysis been performed appropriately and rigorously?

Reviewer #1: N/A

Reviewer #2: N/A

4. Have the authors made all data underlying the findings in their manuscript fully available?

The [PLOS Data policy](http://www.plosone.org/static/policies.action#sharing) requires authors to make all data underlying the findings described in their manuscript fully available without restriction, with rare exception (please refer to the Data Availability Statement in the manuscript PDF file). The data should be provided as part of the manuscript or its supporting information, or deposited to a public repository. For example, in addition to summary statistics, the data points behind means, medians and variance measures should be available. If there are restrictions on publicly sharing data—e.g. participant privacy or use of data from a third party—those must be specified.

Reviewer #1: Yes

Reviewer #2: Yes

5. Is the manuscript presented in an intelligible fashion and written in standard English?

PLOS ONE does not copyedit accepted manuscripts, so the language in submitted articles must be clear, correct, and unambiguous. Any typographical or grammatical errors should be corrected at revision, so please note any specific errors here.

Reviewer #1: Yes

Reviewer #2: Yes

6. Review Comments to the Author

Please use the space provided to explain your answers to the questions above. You may also include additional comments for the author, including concerns about dual publication, research ethics, or publication ethics. (Please upload your review as an attachment if it exceeds 20,000 characters)

Reviewer #1: Thank you for attempting to answer my questions and make the corresponding changes.
One minor comment, when referring to the "nearest-neighbor algorithm" (e.g. in line 153), be aware that this is different (compared to the bilinear interpolation method used in the paper, i.e. has a lower degree (<https://en.wikipedia.org/wiki/Nearest-neighbor_interpolation>).

Response：

Thanks for your exhaustive advice. I'm sorry that I didn't make this part clear. As you said, the "nearest-neighbor algorithm" is different from to the bilinear interpolation method, And the former has a lower degree. The purpose of the nearest neighbor algorithm mentioned in this paper is to reflect the advantages of the bilinear interpolation method in calculation accuracy. We have reorganized this section in the paper.

Reviewer #2: The authors have made substantial changes to the manuscript in this review round and addressed most of my comments. I appreciate the effort of adding the supplemental document to describe the detail of their program.

There's one comment missed. Fig 4(a) still lacks the unit of the data and the author didn't show where they set the boundary condition. In which area the holder is contact with piezoelectric ceramics? It’s recommended to highlight the contact region with some symbol, add the unit of the data, and remove the file path at the bottom of this figure. Meanwhile, please rephrase the caption of Fig 4(b).

Response：

Thank you for your reminding. I'm sorry for the missing comment. The data unit of the Fig 4(a) has been added. We highlighted the location of boundary conditions with black triangle mark, the contact region with red diamond box, and the applied load with red arrow the in Fig 4(a). The file path at the bottom of this figure has also been removed.

The unit of stress in Fig 4(a) is Pascal (Pa). The bottom surface of the hinge and the top and side of the bolt counterbore are fixed according to the actual working conditions. The load is applied on the contact surface, which is highlighted by a red rectangle, between the piezoelectric ceramic front face and the moving block of the hinge.

The caption of Fig 4(b) has been renamed with first natural frequency.

7. PLOS authors have the option to publish the peer review history of their article ([what does this mean?](https://journals.plos.org/plosone/s/editorial-and-peer-review-process#loc-peer-review-history)). If published, this will include your full peer review and any attached files.


If you choose “no”, your identity will remain anonymous but your review may still be made public.


**Do you want your identity to be public for this peer review?** For information about this choice, including consent withdrawal, please see our [Privacy Policy](https://www.plos.org/privacy-policy).

Reviewer #1: No

Reviewer #2: No
